# Supplementary material for: Transcriptomic analysis reveals the impact of concurrent, resistance, and endurance training on skeletal muscle
Source: PLoS One. 2026 Feb 4;21(2):e0340309. doi: 10.1371/journal.pone.0340309 (PMC12871994; doi:10.1371/journal.pone.0340309)
Supplement: Attachment — Submitted filename: Response to Reviewers.docx [file pone.0340309.s004.docx]

**Response to Editor and Reviewer Comments**

**Journal Requirements:**

1. **Style Requirements**: We have reformatted the manuscript using the provided PLOS ONE style templates.
2. **& 3. & 4. Funding Statement**: We have removed all funding-related text from the manuscript. Our updated funding declaration is as follows, and we kindly request you update the online submission form accordingly:
   This study was supported by the University Humanities and Social Sciences Research Project of the Department of Education of Guizhou Province (Grant No. 24RWZX026) and the High-Level Talent Research Start-Up Fund Project of Guizhou Medical University (Grant No. 2024008). Author Longfei Zhao is the recipient of both grants. The funders had no role in study design, data collection and analysis, decision to publish, or preparation of the manuscript.
3. **Data Availability Statement**: We confirm our submission contains the minimal data set to replicate all findings. The primary gene expression dataset analyzed is the publicly available GEO dataset GSE137832. All other data are provided within the manuscript and its Supporting Information files.
4. **ORCID iD**: We confirm that the corresponding author’s ORCID iD has been validated in Editorial Manager.

**Response to Reviewer #1:**

We thank the reviewer for their positive and constructive feedback.

- **Subject Details, Training Protocols, and Physiological Outcomes**: As suggested, we have now included a detailed description of the participants' baseline characteristics (age, height, body mass, BMI), a concise specification of each supervised 12-week training protocol, and the key physiological outcomes (changes in lean body mass, strength, aerobic capacity, anaerobic power) in the revised manuscript (Methods and Results sections).
- **Limitations - Biochemical Analysis**: We appreciate this insightful comment. The relevant sentence in the Limitations section has been amended from "direct histochemical staining" to "**biochemical analysis of myosin isoform expression**."

All changes have been made in the revised manuscript file. We believe these revisions have strengthened the manuscript and thank the editor and reviewer for their time and valuable input.
